# A High-Throughput Microfluidic Platform for Mammalian Cell Transfection and Culturing

**DOI:** 10.1038/srep23937

**Published:** 2016-03-31

**Authors:** Kristina Woodruff, Sebastian J. Maerkl

**Affiliations:** 1 Institute of Bioengineering, School of Engineering, École Polytechnique Fédérale de, Lausanne, Switzerland

## Abstract

Mammalian synthetic biology could be augmented through the development of high-throughput microfluidic systems that integrate cellular transfection, culturing, and imaging. We created a microfluidic chip that cultures cells and implements 280 independent transfections at up to 99% efficiency. The chip can perform co-transfections, in which the number of cells expressing each protein and the average protein expression level can be precisely tuned as a function of input DNA concentration and synthetic gene circuits can be optimized on chip. We co-transfected four plasmids to test a histidine kinase signaling pathway and mapped the dose dependence of this network on the level of one of its constituents. The chip is readily integrated with high-content imaging, enabling the evaluation of cellular behavior and protein expression dynamics over time. These features make the transfection chip applicable to high-throughput mammalian protein and synthetic biology studies.

Reverse transfection assays have the potential to analyze the entire proteome in the natural cellular context. Unlike protein microarrays, this method does not require individual purification of each protein^1^,^2^,^3^. Instead, reverse transfection arrays utilize purified cDNA samples^4^. The array is seeded with cells, which upon transfection convert the cDNA into protein. Arrays can also be made of siRNA to perform loss-of-function studies. With a pitch of approximately 400 μm, more than 5,000 samples can be printed on a single glass microscope slide^4^,^5^. A recent study was able to further increase this density, printing spots just 150 μm apart^6^. This throughput is especially important in light of ongoing efforts to screen genome-wide RNAi libraries^7^ and cDNA libraries^8^. With the accumulation of data from cDNA microarrays and whole genome sequencing, there is a need to validate protein function and characterize therapeutic targets.

Reverse transfection could also serve as a valuable tool for synthetic biology. Synthetic biology is often performed in prokaryotic models because of the ease with which prokaryotes can be genetically modified and interrogated. Engineering mammalian systems remains more difficult, but mammalian synthetic biology is posed to impact biotechnology such as protein production and provide novel therapies through stem cell engineering^9^,^10^. However, the lack of high-throughput tools to efficiently deliver genetic material to mammalian cells is slowing down progress. Mammalian synthetic systems can be complex and require multiple constructs to be simultaneously delivered at precise ratios, necessitating painstaking optimization of the transfection conditions^11^,^12^. Reverse transfection could provide a solution to this problem, but to date it has not been adapted for this purpose.

Although reverse transfection has applications in many fields and is easily scalable, the method involves manual cell seeding and culturing. Moreover, spots on the live cell array are not physically separated from one another. These conditions preclude precise control over the cell environment and increase the likelihood of cross-contamination, to the extent that attempts have been made to separate DNA spots with silicon gaskets and cell-repellent coatings^13^,^14^. The integration of transfected cell arrays with microfluidics could eliminate these concerns, enabling long-term experiments and studies using poorly adherent or highly migrant cell types. Nevertheless, microfluidic transfection devices have yet to reach the impressive throughput of the original reverse transfection microarrays. In a recent method developed by Schudel *et al.* a separate microfluidic channel was required to introduce each lipid-DNA sample, allowing a maximum of 8 unique transfections to be performed on chip^15^. In another study, a lipid-DNA array was generated and aligned to an 8-chamber chip^16^. However, this system was not designed to replenish medium in the transfection chambers, thus prohibiting long-term experiments or flow manipulation.

In this article, we present a platform that combines reverse transfection with microfluidics. Up to 280 independent transfections can be performed per chip, with transfection efficiencies of up to 99% and minimal cross-contamination. The use of a microarrayer to deposit DNA constructs significantly increases throughput, while the microfluidic environment permits transfection, long-term culturing and manipulation of transfected cells. The setup can be continuously imaged, enabling time-lapse studies. We thoroughly characterized our new integrated microfluidic reverse transfection array and applied it to synthetic gene circuits.

## Results

### Device Design and Cell Loading/Culturing

**Figure 1:**
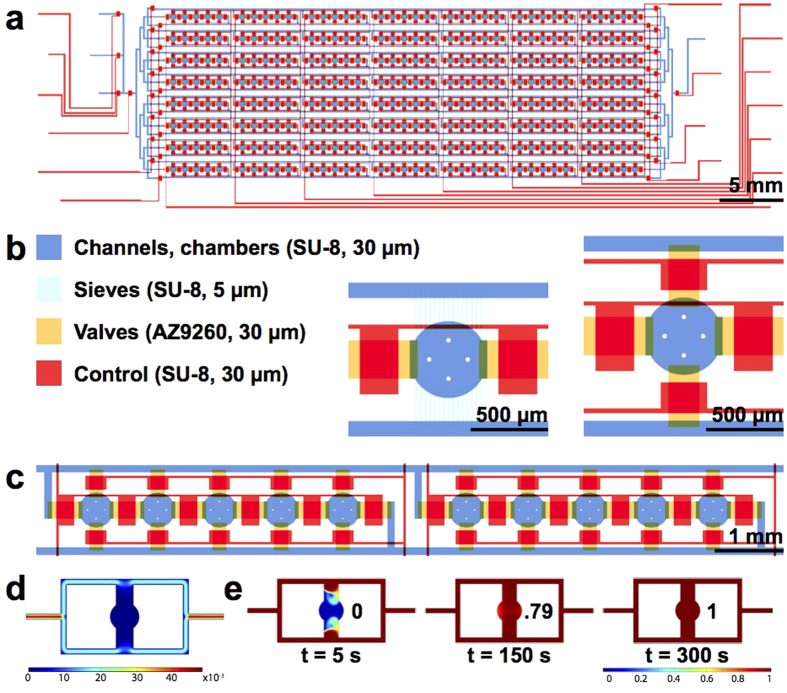
High-throughput microfluidic cell culturing and transfection chip. (**a**) Schematic of the 280-chamber valve perfusion chip. (**b**) The different layers of the device were patterned in photoresist. Close-up images show the sieve design (left) and the valve design (right). (**c**) The chip is loaded in segments of 5 chambers. (**d**) COMSOL model showing the flow velocity profile of the valve design (Scale: m/s). (**e**) COMSOL model showing the diffusion of nutrients into the center of the chamber using a 13.4 kDa protein with a diffusion coefficient of 1.14 × 10^−6^ cm^2^/s  as an example. The extent of diffusion (0–1) is indicated.

Standard reverse transfection has been used by various groups with transfection efficiencies ranging from 13 to 80%^17^,^18^,^19^. We sought to create a highly reproducible and efficient reverse transfection microfluidic platform that supports more complex experiments. Our high-throughput chip design (Fig. 1a, Supplementary Fig. 1a) measures 1.6 × 5.8 cm and contains 280 cell-culturing/transfection chambers. A low-throughput chip containing 80 chambers was also used (Supplementary Fig. 1b). The chips are aligned to DNA arrays so that each of the cell chambers contains a unique transfection reaction. Cell loading takes no more than 10 min and consists of two steps: first, a suspension of HEK 239T cells is flowed through the channels, and second, the channels are segmented into individual chambers by valves (Supplementary Fig. 2). Up to 600 cells can be cultured in each chamber (diameter: 500 μm and height: 30 μm; Fig. 1b).

To achieve both high cell viability and uniform cell density throughout the chip, we loaded segments of the chip sequentially and at low flow velocity. In contrast, an un-segmented chip requires high loading speed to prevent clogging, and this velocity has been shown to decrease cell viability^20^. Our layout permits the loading of 5 columns at a time (Fig. 1c). Each of these subsections can be addressed individually, making it possible to load the device with different cell densities or even different cell types. A loading rate of 7.2 μl/min (volume of medium exiting the chip over time) is used for the low-throughput chip. Due to an increased number of features, the high-throughput chip requires a flow rate of 27.3 μl/min in order to achieve the same linear velocity through the channels.

Cell culturing is performed in a shear-free manner to reduce cross-contamination, minimize cell stress, and increase compatibility of the device with weakly or non-adherent cells. Although shear-free microfluidic perfusion systems have been designed^21^,^22^,^23^, most are limited in throughput because chambers are not separated from one another. In our 80 and 280-chamber devices, we perfused medium at a rate of 8–17 μl/min through supply channels that run parallel to the cell chambers, eliminating sheer stress (Fig. 1a). 5 μm high sieves or 30 μm high valves connect the flow channels and culturing chambers, preventing cell escape from the chambers during medium perfusion (Fig. 1b). Diffusion through the sieves or valves (when opened) introduces nutrients and eliminates waste from the chambers.

COMSOL modeling verified that flow through the chambers was nonexistent (Fig. 1d). According to the model, near-complete diffusion of medium from the side channels to the center of the chambers occurs after 5 h for the sieve design (Supplementary Fig. 1c) and 5 min for the valve design (Fig. 1e). Since we were working with adherent HEK 239 T cells, we chose to use the valve design for most experiments. After pulse perfusing^24^ (see methods) for one hour, cells adhere inside the chambers and the valves can remain open for optimal diffusion of nutrients. Nevertheless we also tested transfection with the sieve chip, which can be used for weakly or non-adherent cells without the need for a pulse perfusion system. We found that due to limited diffusion into the chambers of the sieve chip, the FBS used to prepare the culture medium should be as fresh as possible (Supplementary Fig. 3).

### Device Assembly

Initial microfluidic cell culturing experiments were performed using low-throughput PDMS chips that were bonded to glass slides by baking at 80 °C. For high-throughput chips with densely packed features, the decreased surface area of PDMS available for bonding resulted in frequent delamination of the chip from the glass. To resolve this issue, we investigated whether oxygen plasma could be used to mediate binding between PDMS and DNA-glass arrays. To our knowledge, oxygen plasma has not been used on glass slides patterned with biological substances such as lipid-DNA complexes because the oxygen plasma could potentially destroy the spotted material. We thus tested the effect of plasma on DNA arrays that were completely exposed to plasma and also on arrays where the DNA spots were protected with a PDMS block (Supplementary Fig. 4). The plasma-treated arrays were seeded with cells and measured for transfection efficiency. We found that 7 s plasma treatment, with or without the protective PDMS block, did not affect the spotted DNA, as transfection efficiency was comparable to an array that was not plasma treated (Supplementary Fig. 4). The strong glass-PDMS bond resulting from plasma treatment can sustain increased medium perfusion speeds and drastically enhances the structural stability of the assembled device, which is critical for performing long-term experiments.

### Generating a microfluidic-compatible reverse transfection array

**Figure 2:**
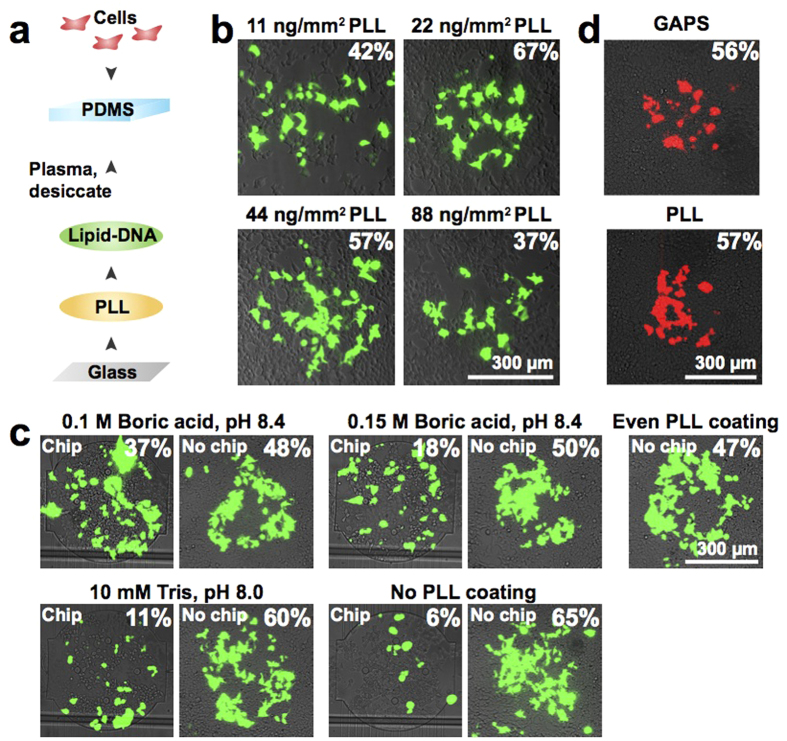
Generation of a microfluidic-compatible reverse transfection array. (**a**) Workflow to fabricate the transfection device. (**b**) Optimization of the amount of PLL deposited during microarraying. eGFP transfection efficiency is indicated for each composite fluorescence image. (**c**) Effect of the composition of the PLL spotting mixture on eGFP transfection efficiency. The images on the left of each set represent the full assembly (PLL spotted array + DNA array + chip). The images on the right of each set represent the assembly without the chip (PLL spotted array + DNA array). A standard reverse transfection array (evenly coated PLL + DNA array) was also tested. eGFP transfection efficiency is indicated for each composite fluorescence image. (**d**) Comparison of tdTomato transfection efficiency using a standard GAPS slide or our spotted PLL slide. Transfection efficiencies are indicated for the composite fluorescence images.

Reverse transfection requires an arraying substrate that retains DNA, such as gamma-amino propyl silane (GAPS) or poly-l-lysine (PLL). However, these surfaces are positively charged and do not bind robustly to hydrophobic PDMS microfluidic chips. To develop a PDMS-compatible substrate, we first arrayed PLL, followed by arraying lipid-DNA complexes onto the PLL spots (Fig. 2a). Arraying of PLL and lipid-DNA is automated and takes between 30 min and 2 h to complete.

Depending on the exact application, PLL-coated glass slides are fabricated using solutions containing 25 μg/ml to 1 mg/ml PLL and various buffers^25^,^26^,^27^,^28^,^29^. Due to the large discrepancies in protocols, we tested a range of PLL concentrations for microarraying (Fig. 2b). PLL arrays were spotted with a standard lipid-DNA mixture and seeded with cells to test for transfection efficiency. We obtained optimal results when depositing a 333 μg/ml PLL solution 4 times per spot, resulting in spots containing ~22 ng/mm^2^ of PLL (Fig. 2b). The time delay between each of the 4 spotting cycles did not considerably alter transfection efficiency (Supplementary Fig. 5a). Arrays were washed after arraying to remove excess PLL, and the time elapsed between array completion and washing did not significantly affect array quality (Supplementary Fig. 5b).

We next optimized the chemical composition of the PLL spotting solution, testing recipes derived from oligonucleotide microarraying protocols as well as tissue adhesion protocols^29^,^30^ (Fig. 2c). To examine the differences between standard and microfluidic transfection, we ran a control array that lacked the microfluidic chip. Arrays made with a water-PBS mixture that is typically used to prepare microarrays yielded very low transfection efficiency. When we spotted PLL diluted in pH-adjusted 0.1 M boric acid, we achieved moderate transfection efficiency on chip (37%) that corresponded well to the efficiency of the control array lacking a chip (48%) (Fig. 2c). These results were also comparable to the 47% efficiency obtained when spotting DNA on an evenly coated PLL slide, which is the standard substrate for reverse transfection.

In addition to identifying the optimal buffer-PLL mixture for on-chip transfection, these experiments also revealed differences in the nature of on-chip vs. standard array setups. The control array lacking a chip yielded efficiencies of 48–65% for all PLL mixtures spotted (Fig. 2c). On chip, depending on which buffer-PLL mixture was used, transfection efficiency ranged from 11 to 37% (Fig. 2c). There was also a stark difference between the chip and control arrays for positions where no PLL was deposited beneath the lipid-DNA. On the control array, this spot yielded a transfection efficiency of 65% (Fig. 2c). In contrast, the same array aligned to a chip had just 6% efficiency (Fig. 2c). These findings indicate that the presence of PLL is crucial for the success of on-chip reverse transfection.

We also tested our optimized PLL arrays alongside commercial GAPS (gamma-amino propyl silane) slides, which are the standard substrate for reverse transfection, and found that both PLL and GAPS substrates performed equally well (Fig. 2d). Compared to the manual batch coating method, our PLL arraying method is simple and precise because an automated microarrayer is used to deposit the PLL. This method is also cost-effective since it requires 100 times less PLL than traditional coating. Moreover, when optimized, in-house coated PLL slides have been shown to support higher and more consistent DNA retention than commercially available slides^31^.

### Microarraying and composition of transfection complexes

**Figure 3:**
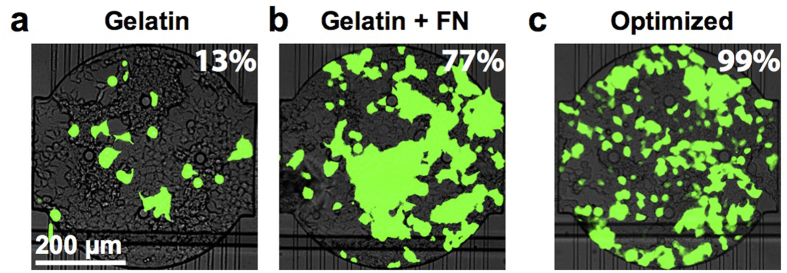
Optimization of the lipid-DNA composition. (**a**) Microfluidic transfection arrays were prepared using mixtures containing eGFP plasmid DNA, Effectene, and gelatin. eGFP transfection efficiency is indicated for the composite fluorescence image. (**b**) As in (**a**), but including fibronectin in the transfection mixture. (**c**) As in (**a**), but using a higher DNA concentration and an optimized PLL array.

After optimizing the PLL arraying, we optimized transfection efficiency by varying the composition of the lipid-DNA mixture. We first used the standard gelatin-containing recipe to prepare lipid-DNA complexes for reverse transfection^4^,^18^,^19^,^32^. When this mixture was deposited on the PLL arrays and used in combination with a PDMS chip, 13% efficiency was achieved (Fig. 3a). We next included fibronectin, which has been previously reported to increase transfection efficiency^33^. When used with the PLL arrays, the fibronectin-containing transfection mixture resulted in 77% efficiency (Fig. 3b, Supplementary Table 1). When the gelatin-fibronectin mixture was used in combination with optimized DNA concentration and PLL arrays, we were able to obtain a very high transfection efficiency of 99% (Fig. 3c, Supplementary Table 1). Moreover, different types of cells can be cultured and transfected on the chip. Using an array optimized for HEK cells, we were able to transfect CHO cells at a rate of 25% (Supplementary Fig. 6a). The transfection efficiency can likely be improved by optimizing the protocol for specific cell types.

In addition to generating lipid-DNA arrays, we also tested the gelatin-DNA reverse transfection technique. For this method, we deposited spots containing a mixture of DNA, gelatin, and fibronectin. Effectene transfection reagent was introduced on chip, and transfection complexes were allowed to form before cells were loaded. This method yielded high efficiency of 77% (Supplementary Fig. 6b) and conserves reagent. For the gelatin-DNA method 25 μl Effectene is required for the entire 280-chamber chip or 90 nl per sample, whereas the lipid-DNA method requires 5 μl per sample spotted. A similar amount is consumed by standard reverse transfection arrays, while well plate transfections require significantly more reagent (Supplementary Table 2). Moreover, well plate transfection requires three reagent mixing steps to be performed separately for each sample and manual addition of the samples to the wells. Microfluidic transfection using the gelatin-DNA method requires just one manual dilution; arraying of the samples and addition of the Effectene is automated. Despite this advantage, existing microfluidic and biochip transfection systems have thus far not taken advantage of the gelatin-DNA method^16^,^34^.

### Quantification of cross-contamination

During culturing of the transfection chip, constant flow of medium parallel to the chambers decreases the probability of cross-contamination or communication between chambers (Fig. 1d). However, cell loading requires direct flow through adjacent chambers (Supplementary Fig. 2), and during this process lipid-DNA complexes could be detached from their original arraying position and deposited in a chamber further downstream. To test this possibility we stained the DNA with fluorescent dye, spotting every other chamber of the array with DNA (Supplementary Fig. 7). One portion of the array was spotted with dye only. The control consisted of an array that was gently immersed in PBS. The test array was aligned to a low-throughput chip and cell loading (flow speed of 5.2 μl/min) was simulated with PBS. For both the control and on-chip PBS washes, the fluorescent DNA spots were still visible on the arrays. Fluorescence was not observed in adjacent positions where DNA was not spotted, indicating that high levels of DNA cross-contamination did not occur. Our PLL arraying procedure is thus sufficient to strongly anchor the DNA to the glass surface.

**Figure 4:**
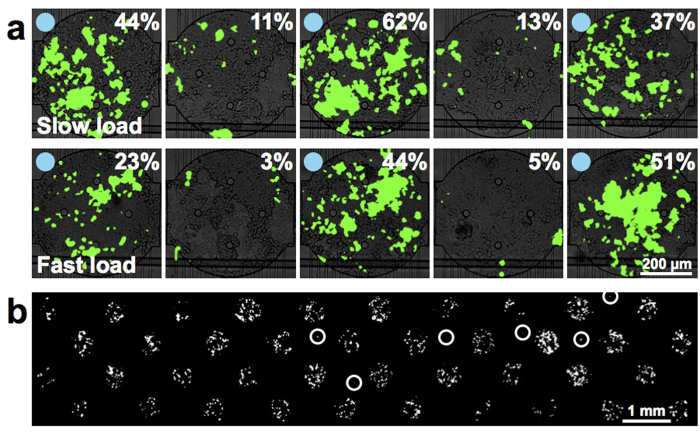
Determination of the source and extent of contamination on the transfection arrays. (**a**) Cells were loaded into a transfection chip either at a slow flow speed of 0.8 μl/min (upper) or a fast flow speed of 5.2 μl/min (lower). Blue circles indicate that DNA was spotted in the chamber. eGFP transfection efficiencies are indicated on the composite fluorescence images. (**b**) Fluorescence micrograph of a chip in which tdTomato transfection mixture was spotted in every other chamber. White circles indicate contaminating cells.

Although these findings imply that lipid-DNA does not detach in significant quantities from the arrays, these experiments were performed in PBS without cells. It is possible that cells take up lipid-DNA complexes as they traverse upstream chambers and ultimately settle in chambers further downstream. To test this possibility we generated a low-throughput array in which every other chamber was spotted with eGFP DNA. When loading at a low speed of 0.8 μl/min, average transfection efficiency across all chambers was 39% in the DNA-spotted chambers and 12% in the non-spotted chambers (Fig. 4a, Supplementary Fig. 8). Loading at a higher speed of 5.2 μl/min resulted in transfection efficiency of 33% in the DNA-spotted chambers and 5% in the non-spotted chambers. It is likely that at slow loading speeds, cells have more time to interact with the DNA spots and acquire more DNA while in transit. Using a loading speed of 7.2 μl/min we obtained extremely low cross-contamination when every other chamber was patterned with tdTomato DNA (Fig. 4b). Of the 40 chambers not patterned with DNA, 6 were contaminated. Two chambers contained two tdTomato-expressing cells, and the other 4 chambers contained one tdTomato-expressing cell.

**Figure 5:**
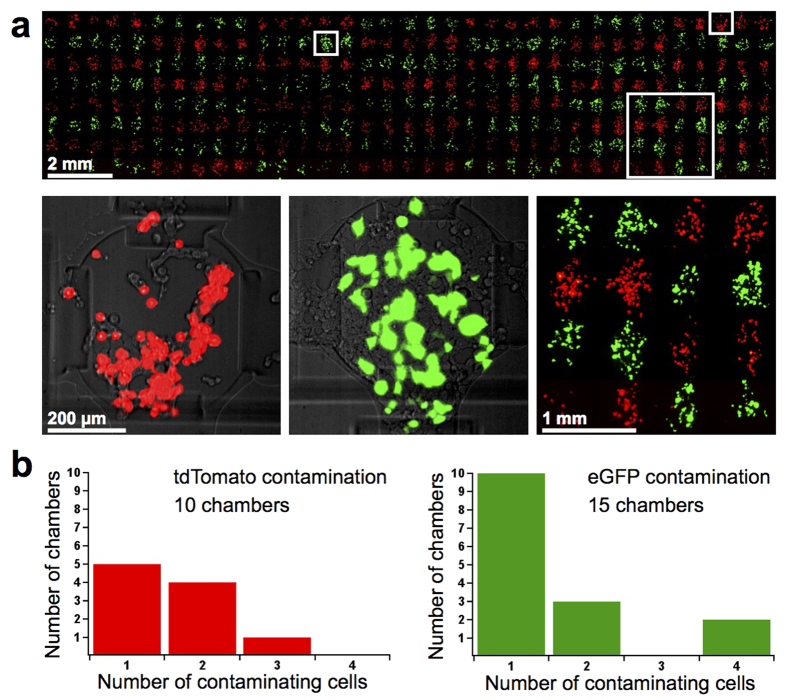
Transfection on the high-throughput chip. (**a**) Fluorescence micrograph of the entire transfected 280-chamber chip (upper) and close-up (lower right). Close-up composite fluorescence images indicate tdTomato and eGFP transfection efficiencies. (**b**) Histograms showing the extent of contamination on the transfected high-throughput chip. eGFP counts were calculated used intensity-adjusted images.

We also performed transfection on the high-throughput chip, transfecting each cell-loading segment with either eGFP or tdTomato. We observed that all 280 chambers were transfected, and at similar efficiencies (Fig. 5a, Supplementary Fig. 9). Average transfection efficiency for all chambers on the chip was 65% (Supplementary Fig. 9). 10 eGFP chambers were contaminated with 1–3 tdTomato cells (Fig. 5b). eGFP contamination in tdTomato-spotted chambers was slightly higher. However, the expression level of eGFP in the contaminating cells was extremely faint compared to the normal level of protein expressed by a transfected cell (Supplementary Fig. 10a). The eGFP contamination histogram shown in Fig. 5b has been adjusted to reflect this (Supplementary Fig. 10b). The adjusted count reveals that 15 chambers were contaminated by eGFP cells, the majority of which contained a single cell.

### Simultaneous delivery of multiple plasmids

Massively parallel transfection on chip should be useful for optimizing and characterizing synthetic systems implemented in mammalian cells. Sophisticated gene circuits contain many components placed on several plasmids that should be co-expressed at different levels, thus the quantities of the different plasmids must be extensively and painstakingly optimized^12^,^35^. To determine if our device could be used to optimize and characterize synthetic systems, we used our high-throughput chip to transfect cells with tdTomato and eGFP DNA mixed at different ratios. In all experiments the total amount of DNA was kept constant, since it has been well-established that there is a specific concentration of DNA that supports maximal transfection efficiency^36^,^37^. We tested each ratio in a total of 10 chambers, using two 5-chamber segments of the chip (Fig. 1c) and analyzing them separately.

**Figure 6:**
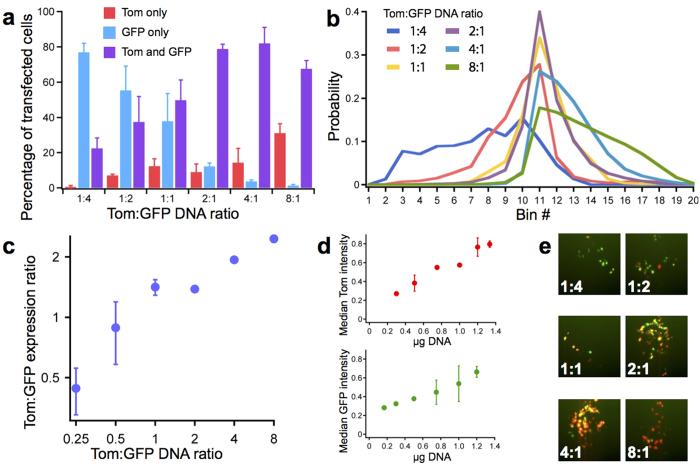
Ratios of protein expression and transfection efficiency during co-transfection on the high-throughput chip. (**a**) Transfection efficiencies as a function of the Tom:GFP co-transfection ratio. Each sample represents the average from 10 chambers patterned with a specific Tom:GFP DNA ratio. Error bars show standard deviation. (**b**) Distribution of Tom:GFP expression ratios. Bin edges span from Tom:GFP expression ratios of 1/6 (left) to 6/1 (right), with increments of 1/0.5 or 0.5/1 (e.g. 1/6, 1/5.5, 1/5 … 5/1, 5.5/1, 6/1). Samples prepared as in (**a**). (**c**) Plot indicating the median Tom:GFP expression ratio from each sample shown in (**b**). Error bars show standard deviation. (**d**) Protein expression (measured by fluorescence intensity) as a function of the amount of DNA transfected. Samples were prepared as in (**a**). Error bars show standard deviation. (**e**) Sample images for each Tom:GFP co-transfection DNA ratio.

First, we evaluated the number of cells that were transfected with eGFP alone, tdTomato alone, or both plasmids. As expected, we found that the transfection efficiency of each plasmid correlated with its concentration in the co-transfection mixture (Fig. 6a). The number of cells expressing both tdTomato and eGFP progressively increased as the Tom:GFP ratio increased from 1:4 to 2:1, reaching a peak of 82% at the 4:1 ratio and decreasing at the 8:1 ratio (Fig. 6a,e). This experimental setup can be used to find the DNA ratio that maximizes the number of cells expressing both plasmids.

Next, we considered only the cells that expressed both proteins and evaluated their protein expression levels by measuring the fluorescence intensities of tdTomato and eGFP. We observed that protein expression ratios were dependent on the ratio of plasmid DNA used (Fig. 6b,c). There was also considerable agreement between the two 5-chamber segments that served as replicates of one another (Supplementary Fig. 11). At more extreme ratios, we observed a weaker correlation between the input DNA ratio and the actual protein expression ratio. For example, the 8:1 sample resulted in a 2.46:1 expression ratio while the 2:1 sample yielded a more predictable value of 1.38:1 (Fig. 6c). Measured protein expression ratios are only relative values and likely skewed from the true values since they are based on measured intensity, and the tdTomato and eGFP fluorophores have different intrinsic brightness. Nevertheless, these findings show that it is possible to tune the expression ratios of proteins inside transfected cells.

When we processed the tdTomato and eGFP data separately, we observed a similar correlation between the amount of DNA used for transfection and the level of protein expression. Increasing the quantity of DNA used for transfection directly increases the fluorescence intensity in a manner that is similar for both tdTomato and eGFP (Fig. 6d). Therefore in addition to co-transfection the transfection device can be used to produce precise levels of a single protein, provided that a second empty plasmid is added to keep the total amount of DNA transfected at the optimal level of 1.5 μg.

These on-chip results corroborated well with the data obtained from performing the same experiment in 96 well and 6 well plate format (Fig. 6, Supplementary Figs 12,13 and 15). In contrast to the reverse transfection array technique used on chip, for the well plates the transfection mixture was added in solution to cells that had already been seeded. The ability of the on-chip results to match the trends displayed by the well plate results assures that this technology can be used interchangeably with standard cell culturing and transfection methods.

In addition to validating that on-chip transfection is comparable to well plate transfection, we also validated our image analysis approach. To do so, we implemented the standard 6 well plate transfection method followed by flow cytometry analysis. The flow cytometry-derived distribution of eGFP-only, tdTomato-only, and co-transfected cells displayed the same dependency on DNA ratios that was seen with the data obtained by image analysis (Supplementary Figs 12,13,15 and Fig. 6). The relationship between co-transfection DNA ratio and protein expression ratio was also similar, with flow cytometry performing better at more extreme ratios (Supplementary Figs 13,15 and Fig. 6). Image analysis thus can serve as a less complicated alternative to flow cytometry, as there is no need to harvest and prepare each cell sample.

### Transfection of synthetic genetic circuits

**Figure 7:**
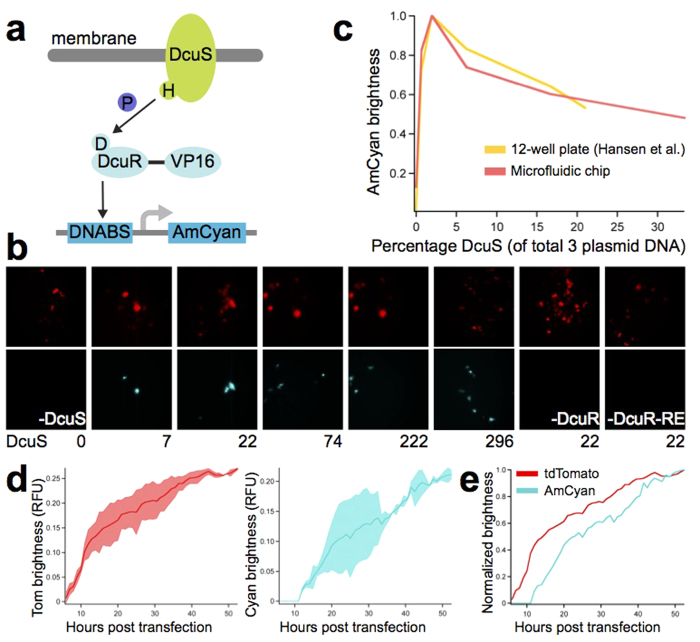
Measuring the dynamics of synthetic gene circuits on the high-throughput chip. (**a**) Schematic of the two-component signaling pathway. The histidine kinase (DcuS) is activated by ligand binding and transmits the signal to DcuR, a DNA-binding protein. P, phosphate; VP16, VP16 transactivator domain, DNABS, DNA binding sites. (**b**) Representative fluorescence images with DcuS amounts indicated in ng. Reactions included 296 ng tdTomato and, aside from the negative controls, 444 ng DcuR and 665 ng DcuR-RE. (**c**) Brightness of the reporter as a function of histidine kinase concentration for the original 12-well setup^35^ and for the microfluidic setup. For the microfluidic data, points are the average of 10 chambers. Brightness is normalized so that the maximum occurs at 1. (**d**) Dynamics of tdTomato and AmCyam protein expression over time. The sample contained 296 ng tdTomato, 59 ng DcuS, 444 ng DcuR 665 ng DcuR-RE. The average brightness of two 5-chamber segments of the chip was calculated separately, and shaded areas represent the standard deviation between these two values. Maximum brightness is 1. (**e**) As in (**d**), but with brightness normalized so that the maximum occurs at 1.

Many different co-transfection ratios can be tested in parallel on the chip, facilitating optimization and enabling high-throughput mammalian synthetic biology. To test the device with a recently reported synthetic system, we implemented the two-component signaling (TCS) system developed by Hansen *et al.*^35^. The pathway consists of prokaryotic proteins DcuS and DcuR^38^,^39^ that have been codon-optimized for expression in mammalian cells and are constitutively expressed from a CMV promoter. Upon stimulation by C_4_-carboxylates present in the cell culture medium, the membrane-localized histidine kinase (DcuS) autophosphorylates its cytoplasmic domain (Fig. 7a). The phosphate is then transferred to DcuR, a transcriptional regulator that has been modified to contain three minimal VP16 transactivating domains at the C terminus. Upon activation by phosphorylation, DcuR binds to the promoter of the reporter plasmid and activates transcription of AmCyan.

Various ratios of the three plasmids were used for transfection on the high-throughput chip, and a fourth plasmid (expressing tdTomato) was used as a transfection control. Activation of AmCyan expression was observed for all ratios tested (Fig. 7b). Transfections lacking any one element of the synthetic circuit did not produce AmCyan fluorescence and only expressed the tdTomato transfection control. We next tested the sensitivity of the system to changes in DcuS while keeping DcuR and the AmCyan reporter at fixed quantities.

We found that maximal AmCyan induction occurs when DcuS represents 2% of the total 3-plasmid transfection mixture and decreases below the optimal DcuS concentration (Fig. 7c). This finding is in agreement with the original study, which implemented transfections in 12-well plates followed by flow cytometry analysis of protein expression. We additionally performed the experiment in 96-well plate format followed by image analysis of protein expression and obtained similar results (Supplementary Fig. 14). Our microfluidic transfection and image analysis method is thus capable of reproducing the results obtained from standard transfection and flow cytometry techniques.

We also performed time-lapse experiments to visualize the changes in protein expression over the course of transfection. Expression of the tdTomato transfection control began immediately post transfection (Fig. 7d,e, Supplementary Movie 1). AmCyan expression was first detected 12 h post transfection (Fig. 7d,e, Supplementary Movie 2). Assuming that tdTomato and AmCyan have similar maturation times^40^,^41^, this time lag can be explained by the fact that DcuS and DcuR must first be transfected and expressed before they can activate expression of AmCyan (Fig. 7a). For both tdTomato and AmCyan, protein expression levels continued to increase steadily until 53 h post transfection (Fig. 7d,e, Supplementary Movies 1 and 2). These results support the standard practice of acquiring images after 48 h, when transfection efficiency and protein expression are high. Because our microfluidic technology is compatible with high-content imaging, samples can be monitored continuously, allowing significantly more data to be collected compared to end-point methods such as FACS.

## Discussion

We developed a high-throughput microfluidic platform capable of 280 independent transfections at up to 99% efficiency with HEK 293 T cells. In comparison, other biochip platforms obtained between 13% and 80% transfection efficiency^17^,^18^,^19^, while throughputs ranged between 1 to 96 reactions per device^15^,^16^,^42^,^43^. The transfection reactions on our chip are confined to separate chambers, decreasing the risks of cross-contamination and communication between different positions on the array. This segregation, combined with the chip’s ability to automate long-term cell culture, has the potential to overcome the limitations of reverse transfection microarrays. Microfluidic platforms are easily adapted to high-content imaging^44^,^45^,^46^, and the reduced sample requirement enables the study of cells that are available in limited quantities. The behavior of cells over time can further be monitored in response to fluidic stimuli such as change of the culturing medium or introduction of a drug^47^. By implementing a recently developed synthetic two-component system we show that our integrated microfluidic transfection platform can be used to optimize and study synthetic circuits. Overall, the high-throughput microfluidic transfection and cell-culturing platform we demonstrate here should be a useful tool for mammalian cell engineering.

## Methods

### Microarray printing and slide treatment

Prior to arraying, glass slides were washed by shaking in a 57% Ethanol, 10% w/v NaOH solution for 2 h. The slides were thoroughly washed with Milli-Q water and dried before proceeding to microarray printing. A QArray2 microarrayer (Genetix GmbH) was used to array samples contained in conical-well, poly(propylene) 384-well plates (Arrayit). Most solutions were spotted using a pin with a 300 μm spot diameter and 3.3 nl delivery volume (946MP9, Arrayit). For the 2-component system, a 500 μm spotting pin was used (946MP15, Arrayit). Spotting parameters for both lipid-DNA mixtures and PLL were 1 s inking time, 500 ms printing time. Between samples, the pin was washed with water for 500 ms and dried for 500 ms. Lipid-DNA was stamped once per spot. As has been previously reported^18^, we found that stamping multiple times per spot resulted in imprecisely localized spots due to spreading of excess DNA. Following microarraying, lipid-DNA arrays were immediately stored in a desiccator.

PLL was stamped 4 times per spot in a cyclic fashion, with approximately 8 min between cycles. 2 h after the arraying was complete, PLL arrays were thoroughly washed with filtered water, dried, and stored in a desiccator. PLL slides were used between 2 and 8 weeks post-coating, since the quality of the PLL has been shown to decrease significantly beyond this time period^31^.

### Microarraying mixture preparation

The PLL sample was prepared by adding 25 μl of 0.1% PLL solution (Sigma) to 50 μl of a solution of 0.225 M boric acid, pH 8.4. Evenly-coated PLL slides were prepared according to standard protocols^29^. The lipid-DNA method developed by Sabatini *et al.*^4^ was used to prepare transfection mixtures containing Effectene (Qiagen). First, 1.5 μg of supercoiled plasmid DNA was diluted in 15 μl of EC buffer in which sucrose had been dissolved at a concentration of 0.2 M. In the case of co-transfection, this mixture was vortexed for 10 s and allowed to incubate for 15 min. Next, 1.5 μl Enhancer was added and the mixture was vortexed for 1 s and incubated at room temperature for 5 min. Next, 5 μl Effectene was added and the mixture was vortexed gently for 10 s and incubated for 10 min. Lastly, 12.7 μl of a 0.5% gelatin solution (prepared from G9391, Sigma) and 12.7 μl of a 0.1% fibronectin solution (F0895, Sigma) were added and the samples were transferred to a 384-well plate for microarraying. Human plasma fibronectin could be replaced with bovine plasma fibronectin (Sigma-Aldrich F4759, powder dissolved in water at 1 mg/ml) with no decrease in transfection efficiency.

Samples for the gelatin-DNA transfection method were prepared similarly and contained a mixture of 1.5 ug DNA and buffer EC (total of 25.4 μl), 12.7 μl gelatin, and 12.7 μl fibronectin. A mixture of 16 μl Enhancer, 150 μl EC buffer, and 25 μl Effectene was flowed on chip for 30 min using the same conditions used for medium perfusion. Medium was flowed on chip for 20 min before cell loading.

### Standard transfections

96-well plate transfections were performed by first seeding each well with 10,000 cells the day before transfection. Each well was transfected with 0.1 μg DNA diluted in EC buffer, 0.8 μl Enhancer, and 2.5 μl Effectene (prepared according to manufacturer’s instructions). Images were acquired and processed in the same manner used for on-chip experiments. 6-well plate transfections were performed by first seeding each well with 200,000 cells the day before transfection. Each well was transfected with 0.4 μg DNA diluted in EC buffer, 3.2 μl Enhancer, and 10 μl Effectene (prepared according to manufacturer’s instructions). Cells were harvested and subjected to analysis by flow cytometry.

### DNA staining

A 100× dilution of YOYO-1 dye (Life Technologies) was prepared in DMSO (Sigma). The YOYO dilution was added to the complete lipid-DNA transfection mixture to result in a final 10,000 fold dilution. The transfection-dye mixture was incubated for 60 min before microarraying. The stained arrays were visualized by using an ArrayWorx scanner (Applied Precision).

### Cell culture

All culture reagents were acquired from Gibco (Life Technologies). Cells were cultured at 37 °C and 5% CO_2_ in Dulbecco’s modified Eagle medium (DMEM) supplemented with 10% FBS and antibiotic-antimycotic. Cells were passaged every 2–3 days using TrypleE express. For on-chip experiments, cells were grown in CO_2_ independent medium supplemented with GlutaMAX, 10% FBS and antibiotic-antimycotic. Microfluidic chips were cultured on the stage of a microscope contained within an incubation chamber (Life Imaging Services) maintained at 37 °C.

### Flow cytometry

48 h after transfection, cells were trypsinized and resuspended in PBS. For analysis, a BD LSRII was used with the following settings: for eGFP, a 488 nm laser and a 525/50 filter with a photomultiplier tube (PMT) voltage of 225, for tdTomato: a 561 nm laser and 585/15 filter with a PMT voltage of 379. 50,000 events were measured per sample.

### Imaging

Imaging of the microfluidic transfection arrays was performed on a Nikon Ti-E Eclipse automated microscope using NIS Elements. Images were acquired with an Ixon DU-888 camera (Andor Technology), using 20x magnification to capture an entire chamber in the field of view. Each chamber was imaged in brightfield and fluorescence mode. Three HC filter cubes were used: TexasRed (HC 562/40, HC 624/40, BS 593) for tdTomato, FITC (HC 482/35, HC 536/40, BS 506) for eGFP, and CFP (HC 438/24, HC 483/32, BS 458) for AmCyan (all from AHF Analysentechnik AG). Images were stitched together using the Grid/Collection Stitching plugin in Fiji^48^. Cells were counted using the “Load Images”, “Crop”, “Identify Primary Objects”, and “Measure Image Area Occupied” modules of CellProfiler^49^ or by using a custom written Matlab script.

Transfection efficiency was calculated as the area occupied by fluorescent cells in the entire chamber (500 μm diameter) divided by the area occupied by cells within the 300 μm diameter spot where the lipid-DNA was deposited. This normalization is necessary because following cell loading into the chambers, only some of the cells have access to the transfection mixture (a 300 μm spot within a 500 μm chamber). Some cells that initially settle in the lipid-DNA area migrate to different parts of the chamber after 48 h (when images are captured), resulting in the dispersed pattern visible in the images.

### COMSOL modeling

COMSOL Multiphysics was used to build a 2D representation of the microfluidic cell culturing chambers. The modules laminar flow and transport of diluted species were used. Cytochrome C, a 13.4 kDa protein with a diffusion coefficient of 1.14 × 10^−6^ cm^2^/s, was used to model the transport of diluted species.

### Microfluidic device fabrication

Microfluidic devices were designed in Clewin (WieWeb software, Netherlands). Two molds were designed: one for the control layer, which contains the valves, and another for the flow layer, which contains the channels and chambers necessary for reagent introduction and cell culturing. The control layer was scaled by 101.5% to account for PDMS shrinkage during curing.

The molds were fabricated using standard photolithography methods. The control layer mold was patterned with SU-8 photoresist (Gersteltec, Switzerland) to a height of 30 μm. The flow layer mold for the sieve chip design (Supplementary Fig. 1a) was fabricated in five steps. First, to create alignment marks, AZ1512 positive photoresist (MicroChemicals GmbH) was spin coated to a height of 1 μm, then exposed and developed. The alignment marks were etched to a depth of 4 μm using inductively coupled plasma. The AZ1512 hard mask was then removed using oxygen plasma. Second, a dummy layer of SU-8 was spin coated to a height of 2 μm, then flood exposed and developed. Third, sieves were created by spin coating SU-8 to a height of 5 μm, followed by exposure and development. Fourth, flow channels were generated by spin coating SU-8 to a height of 30 μm, followed by exposure and development. Lastly, valve regions were patterned by spin coating AZ9260 (MicroChemicals GmbH) to a height of 30 μm, then exposing and developing. The AZ9260 was annealed at 120 °C for 25 s to generate the rounded profile that is required for complete valve closure. The valve chip design (Fig. 1a) was fabricated by performing only steps four and five of the protocol detailed above.

Polydimethylsiloxane (PDMS; Sylgard 184, Dow Corning Corp., USA) was cast onto the molds and multilayer soft lithography techniques were used to assemble the chip^50^. A thick layer of PDMS (5:1 ratio of parts A:B) was poured onto the control layer, whereas the flow layer was spin coated with PDMS (20:1 ratio of parts A:B) with a ramp of 15 s and a spin of 35 s at 650 rpm. The molds were baked for 30 min at 80 °C. The control layer chips were then cut, removed from the mold, and punched with inlet holes. The control layer chips were aligned to the flow layer, and the assembly was baked for 90 min at 80 °C. The aligned devices were then cut, removed from the mold, and punched with inlet and outlet holes. The PDMS chips were bonded to the glass arrays by using 7 s of oxygen plasma treatment, followed by baking for 1 h at 80 °C. Transfection devices were stored in a desiccator prior to use.

### Microfluidic chip operation

The pressures of the flow and control layers of the chip were controlled by using a custom built pneumatic setup. The valves of the control layer were first primed with filtered water at 5 psi. Once all air had been removed from the control lines, the control layer pressure was increased to 22 psi. Medium was flowed through the chip prior to cell loading. A sample containing 800,000 cells in 20 μl PBS was purged through the chip inlets. After purging, the cells were loaded into the first set of columns at a speed of 7.2 μl/min for the low-throughput chip and 27.3 μl/min for the high-throughput chip. Flow rate was determined by measuring the volume of liquid exiting the chip over a period of time. Columns were loaded in sets sequentially, and the chamber-segmenting valves were actuated once loading for each set was complete. For the sieve design, medium was immediately perfused at a rate of 1 ml/h (16.7 μl/min). For the valve design, medium was pulse perfused for the first hour. Medium was flowed with chamber valves closed (5 min) followed by stopping the flow and opening the chamber valves (5 min). After cycling between these two states for the first hour, the chamber valves were opened during continuous medium flow of 1 ml/h. PTFE tubing was used for all fluidic connections because we observed some cell toxicity when using Tygon tubing, as has been previously reported^23^.

## Additional Information

**How to cite this article**: Woodruff, K. and Maerkl, S. J. A High-Throughput Microfluidic Platform for Mammalian Cell Transfection and Culturing. *Sci. Rep.* **6**, 23937; doi: 10.1038/srep23937 (2016).

## Supplementary Material

Supplementary Information

Supplementary Movie 1

Supplementary Movie 2

Supplementary Movie 3
