# Supplementary material for: A High-Throughput Microfluidic Platform for Mammalian Cell Transfection and Culturing
Source: Sci Rep. 2016 Mar 31;6:23937. doi: 10.1038/srep23937 (PMC4814922; doi:10.1038/srep23937)
Supplement: Supplementary Information [file srep23937-s1.pdf]

Supplementary Information

A High-Throughput Microfluidic Platform for Mammalian Cell Transfection and Culturing

Kristina Woodruff and Sebastian J. Maerkl*

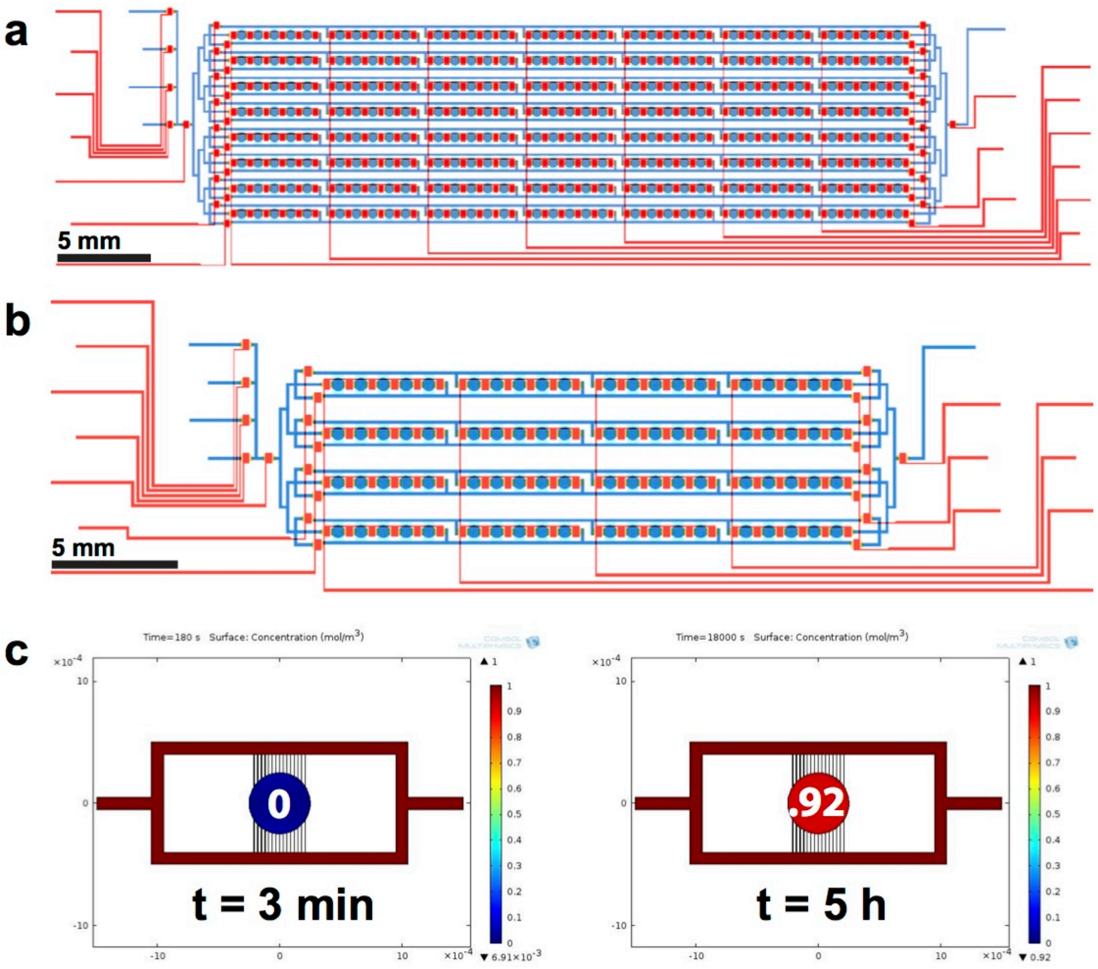

Supplementary Figure 1. Design of the high-throughput cell culturing and transfection chip (sieve version).
(a) Design of the 280-chamber chip with sieves for medium perfusion. The chip measures 1.5 x 5.7 cm. Refer to Fig. 1b for legend. (b) Design of the 80-chamber chip with sieves for medium perfusion. The chip measures 1.2 x 4.1 cm. Refer to Fig. 1b for legend. (c) COMSOL modeling shows that diffusion of nutrients into the center of the cell chamber is 92% complete after 5 h. A 13.4 kDa protein with a diffusion coefficient of 1.14 x 10^{-6} cm^2/s was used as an example.

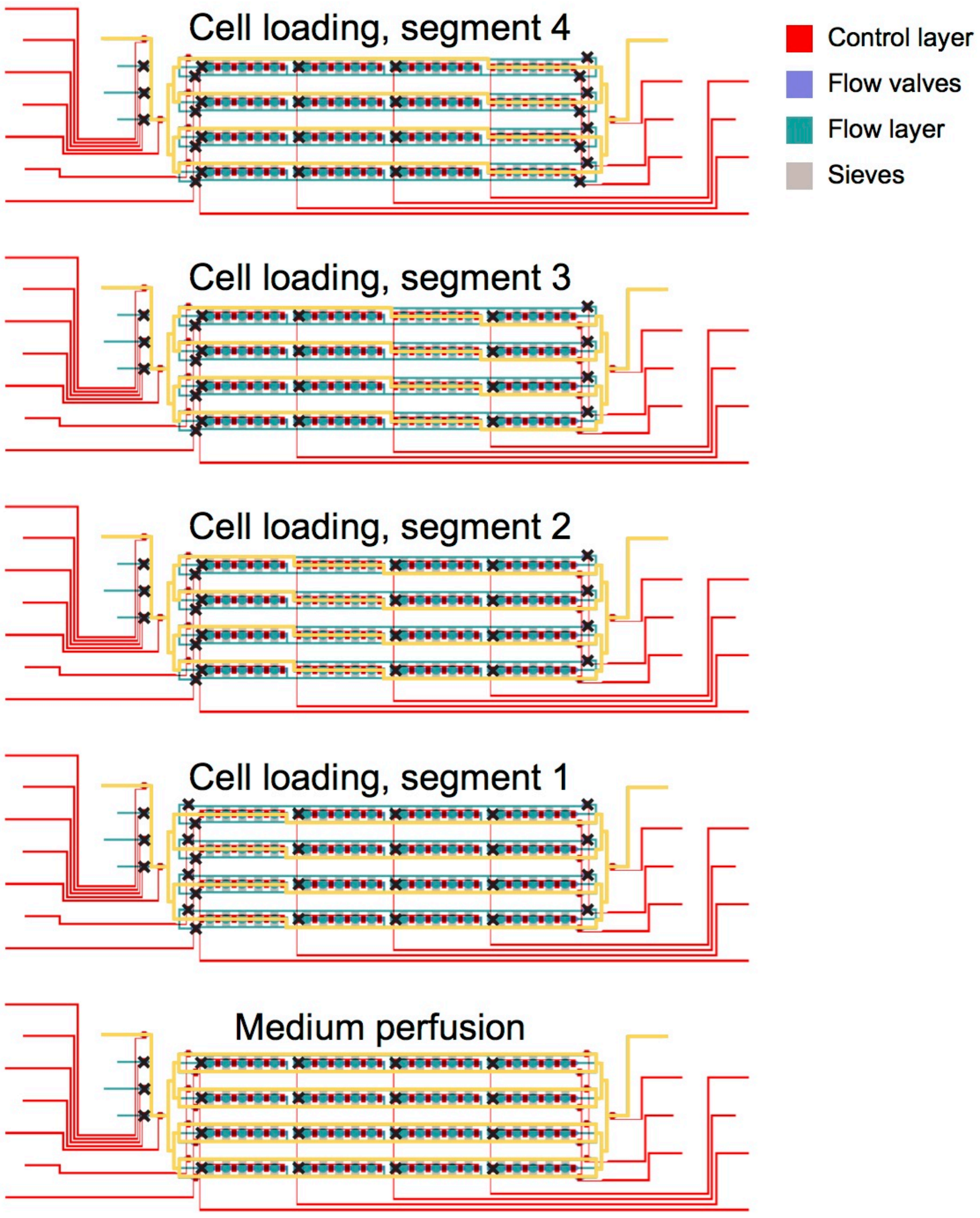

Supplementary Figure 2. Schematic of cell loading and culturing on the low-throughput chip.
Segments of chambers containing 5 columns are loaded sequentially. Yellow indicates the trajectory of flow through the chip. Black crosses indicate valves that are closed. For the chip design containing valves in the place of sieves (Fig. 1b), these valves remain closed during cell loading and are opened during medium perfusion. The high-throughput chip is loaded in a similar manner, the main difference being the number of chamber segments (7 instead of 4).

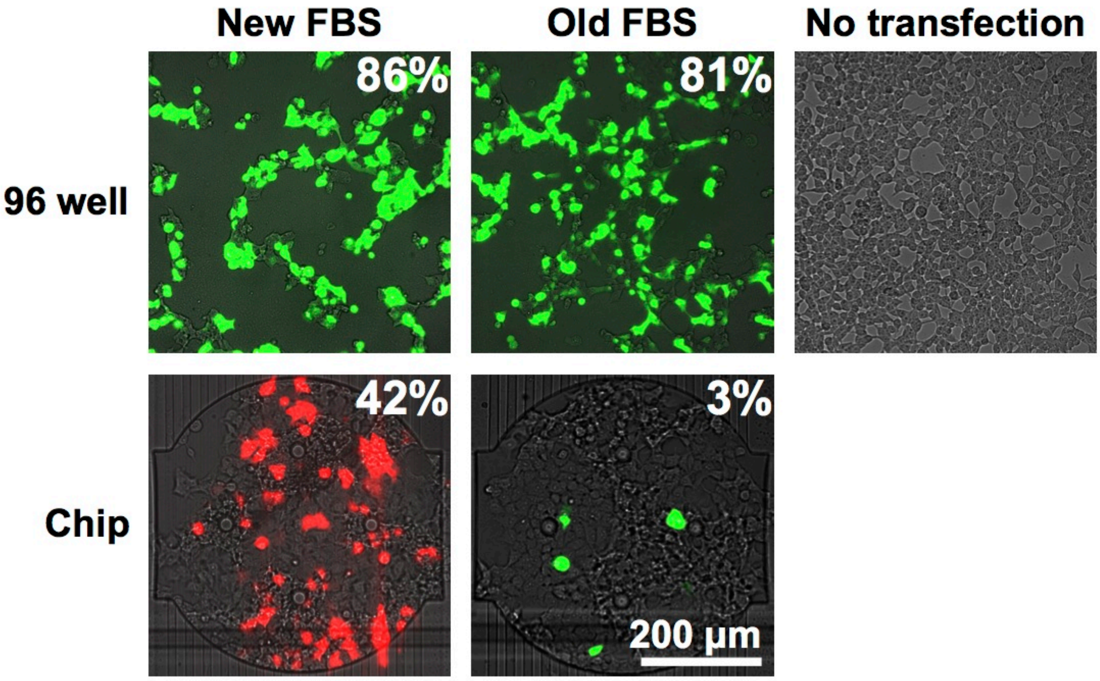

Supplementary Figure 3. The effect of serum quality on transfection efficiency with the sieve device.
Old (freeze-thawed 3 times) FBS was used for transfection experiments on a 96 well plate and on the sieve design chip. Transfection efficiencies of eGFP and tdTomato are indicated in the composite fluorescent images. Cells proliferate faster when no transfection reagent is present.

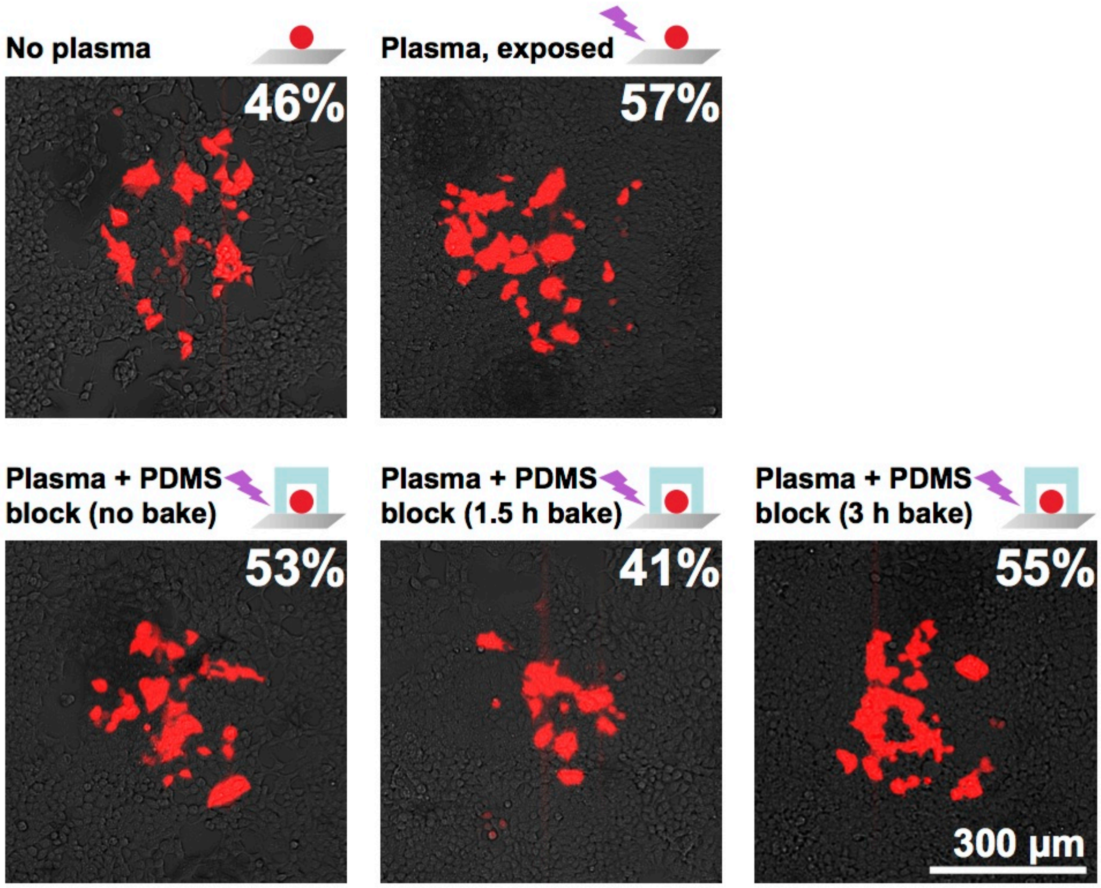

Supplementary Figure 4. Plasma treating of lipid-DNA microarrays. tdTomato transfection arrays were treated with oxygen plasma for 7 s before being seeded with cells. For some arrays, the lipid-DNA spots were protected by a PDMS block that had been adhered to a glass slide immediately before plasma treatment (no bake) or during an 80°C bake (1.5 or 3 h). tdTomato transfection efficiency is indicated for each composite fluorescence image.

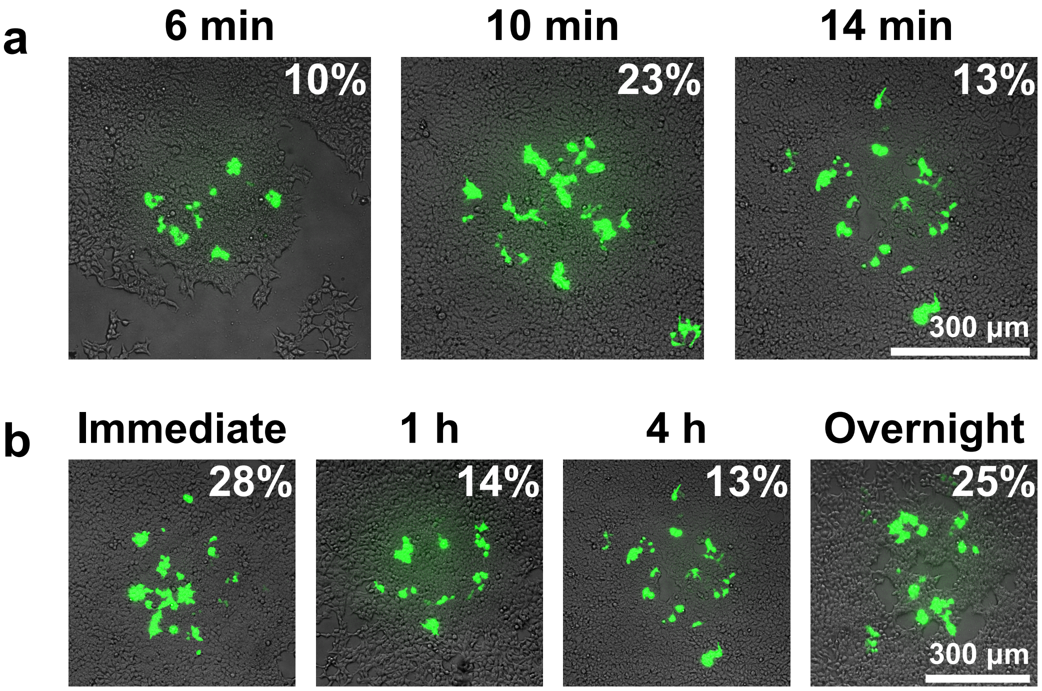

Supplementary Figure 5. Optimization of PLL spotting.
(a) The amount of time elapsed between each of the 4 PLL spotting cycles has little effect on eGFP transfection efficiency, as indicated by the composite fluorescent images. (b) The amount of time elapsed between array completion and array rinsing to remove excess PLL has little effect on eGFP transfection efficiency, as shown on the composite fluorescent images.

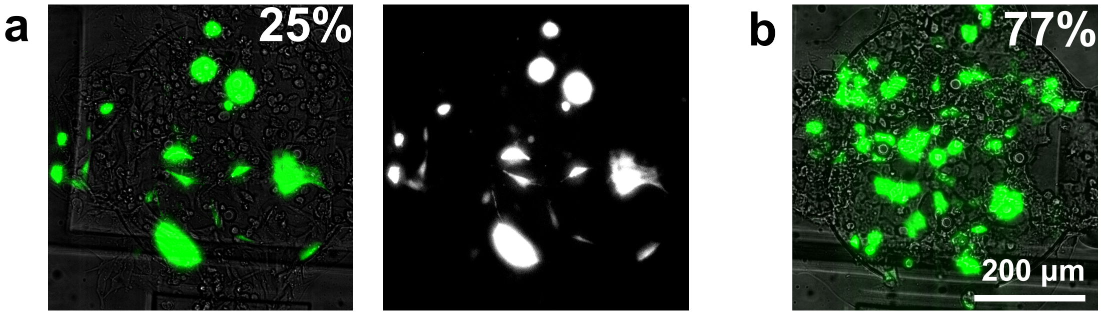

Supplementary Figure 6. Adapting the microchip to different cell types and transfection methods.
(a) CHO cells are transfected with eGFP on chip at high efficiency when using conditions that were optimized for HEK cells. Transfection efficiency is indicated on the composite fluorescent image (left). The fluorescence image is shown on the right. (b) HEK cells are transfected with eGFP on chip at high efficiency when using the gelatin-DNA method. Transfection efficiency is indicated on the composite fluorescent image.

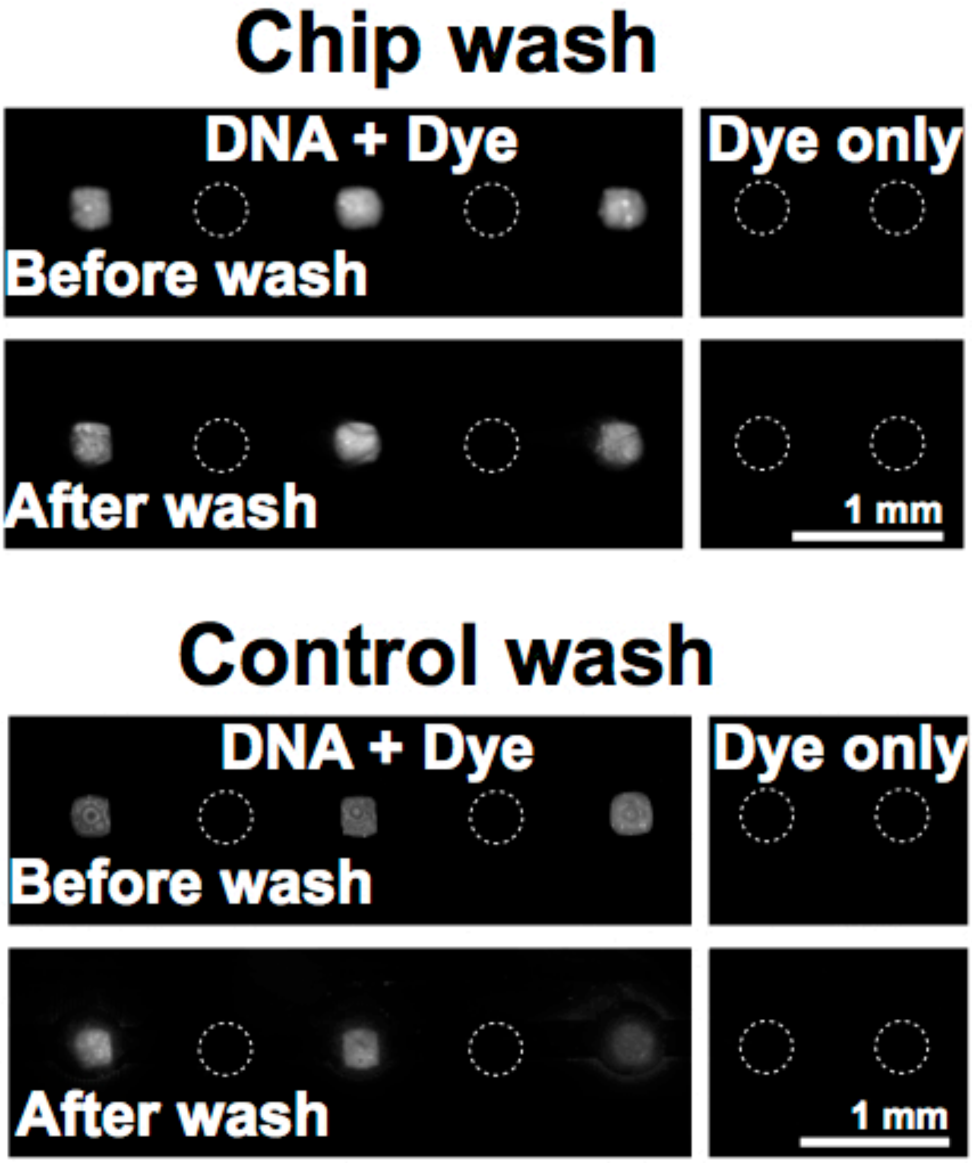

Supplementary Figure 7. Investigation of DNA cross-contamination on the transfection arrays.
Testing DNA cross-contamination by staining with Sybr Green dye. DNA + dye or dye alone were included in the transfection mixture and spotted on a PLL array. Fluorescence scans were obtained before and after washing the arrays with PBS on chip (chip wash) or immersing the arrays with PBS in batch (control wash). Dashed circles indicate positions where nothing (DNA + Dye section) or dye only was spotted. Scale bars, 1 mm.

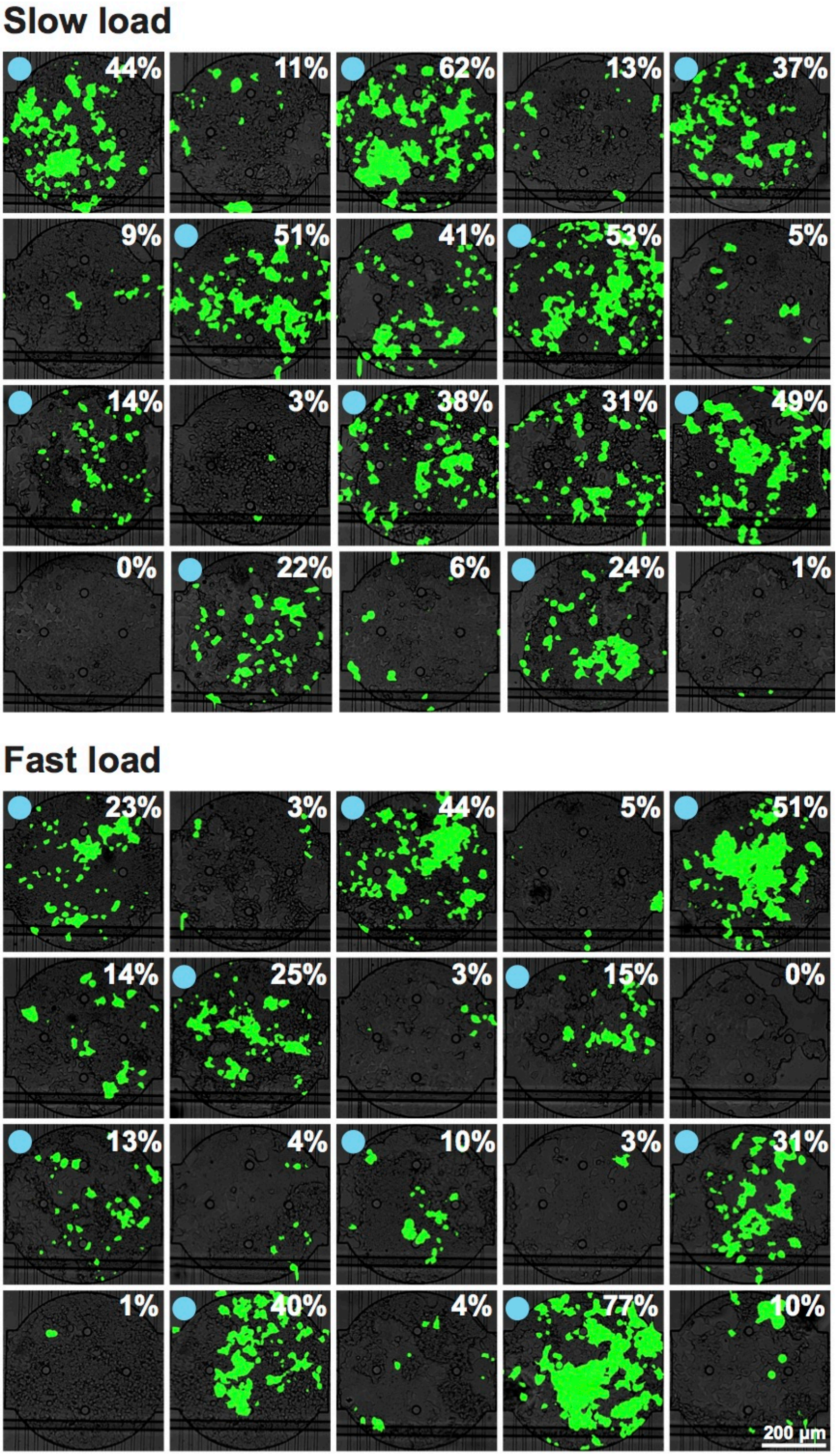

Supplementary Figure 8. Cell loading speed and cross-contamination. The relationship between cell loading speed and cross-contamination was tested using the bottom two rows of a low-throughput chip. The left half of the rows (20 chambers) was loaded at a low speed of 0.8 μl/min, and the right half of the rows (20 chambers) was loaded at a higher speed of 5.2 μl/min. Transfection efficiencies are indicated for the composite fluorescence images. Blue circles indicate that DNA was spotted in the chamber.

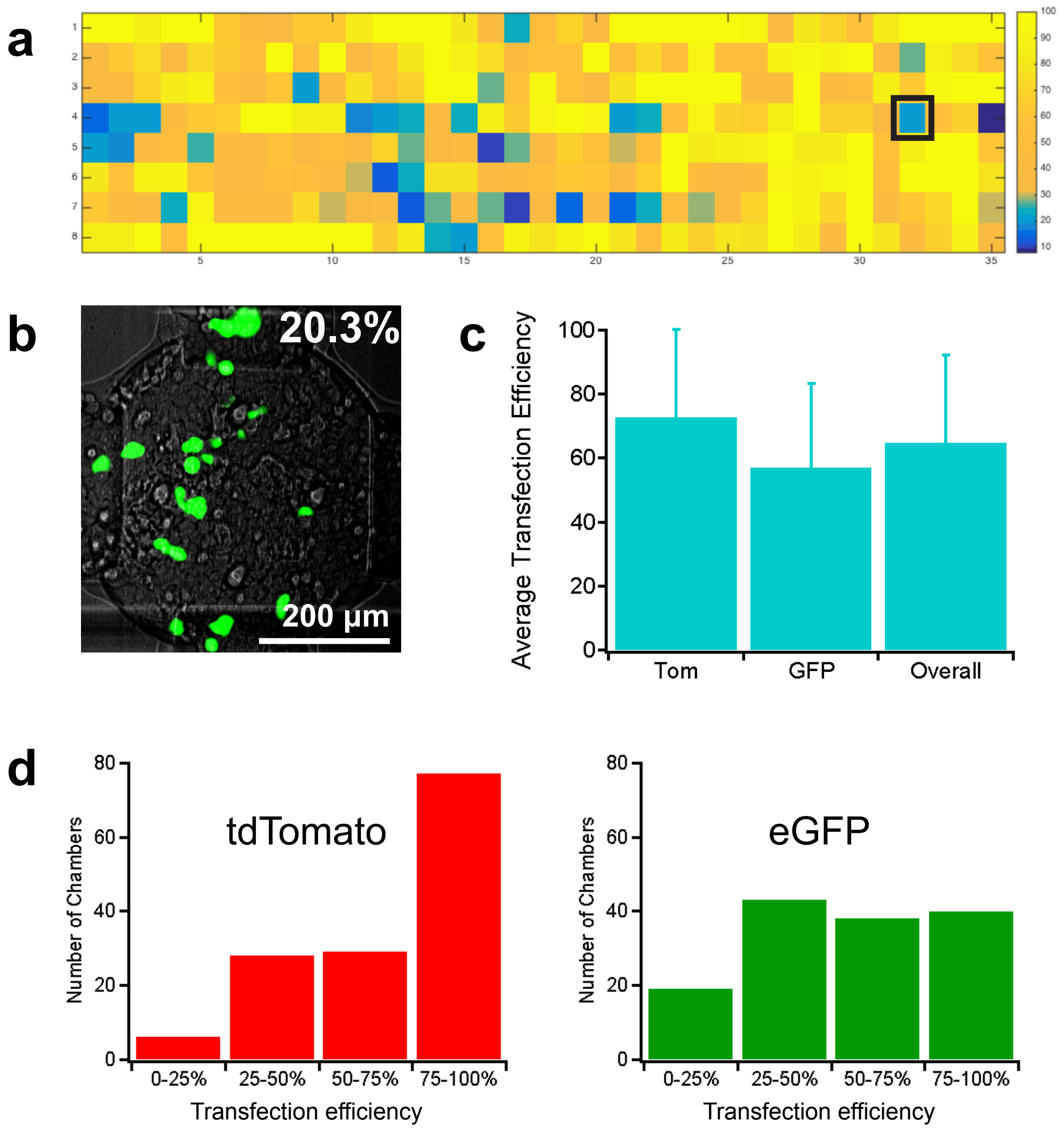

Supplementary Figure 9. Transfection efficiencies on the high-throughput chip.
(a) Heatmap showing the distribution of transfection efficiencies for each chamber of the chip presented in Fig. 5a. (b) Close-up composite fluorescence image of a chamber from (a) with low eGFP transfection efficiency (row 4, column 32). Although a moderate number of cells express eGFP, overall transfection efficiency is low due to the large number of cells in the chamber. (c) Average transfection efficiencies for the chip presented in Fig. 5a. (d) Distribution of transfection efficiencies amongst tdTomato and eGFP chambers.

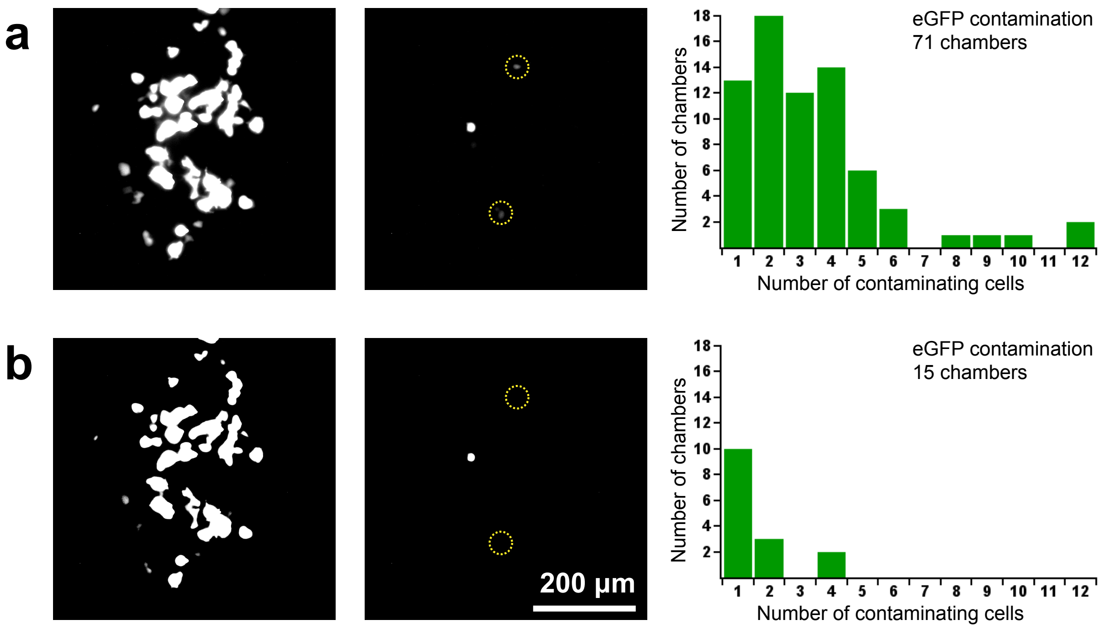

Supplementary Figure 10. Quantification of eGFP contamination on the high-throughput transfection chip.
(a) Unadjusted images showing representative chambers transfected with eGFP (left) and contaminated with eGFP (center) from the chip shown in Fig. 5. Dashed yellow circles indicate the positions of two weakly fluorescent eGFP cells. The histogram on the right indicates the total contamination count when including weakly fluorescent cells. (b) Same as (a), but after adjusting the threshold to exclude weakly fluorescent eGFP cells.

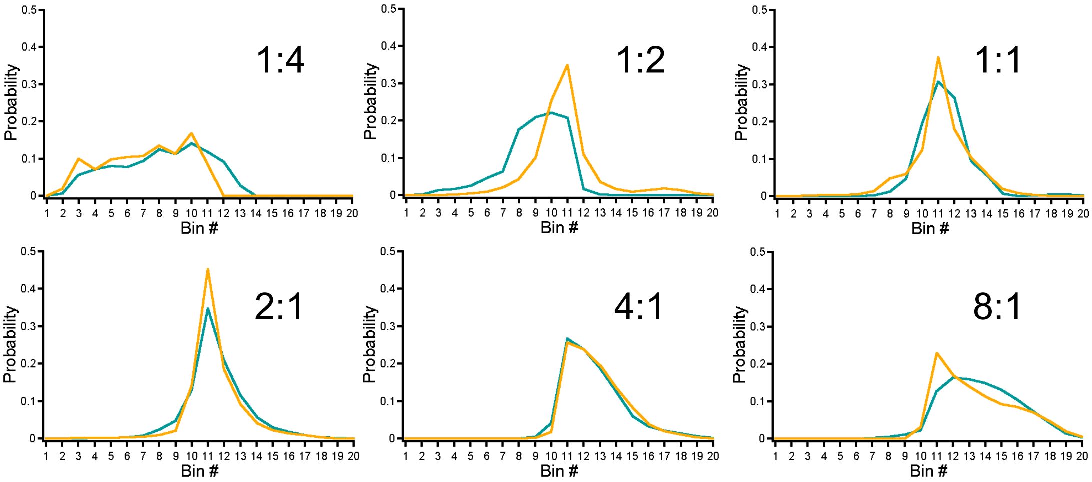

Supplementary Figure 11. Ratios of protein expression and transfection efficiency during co-transfection.
Distribution of Tom:GFP expression ratios for co-transfection ratios ranging from 1:4 to 8:1. Bins span from Tom:GFP ratios of 1/6 (left) to 6/1 (right), with increments of 1/0.5 or 0.5/1 (e.g. 1/6, 1/5.5, 1/5 … 5/1, 5.5/1, 6/1). Each curve represents the averages from one 5-chamber segment of this chip (Fig. 1c). The average of these two data sets was used to generate the curves shown in Fig. 6.

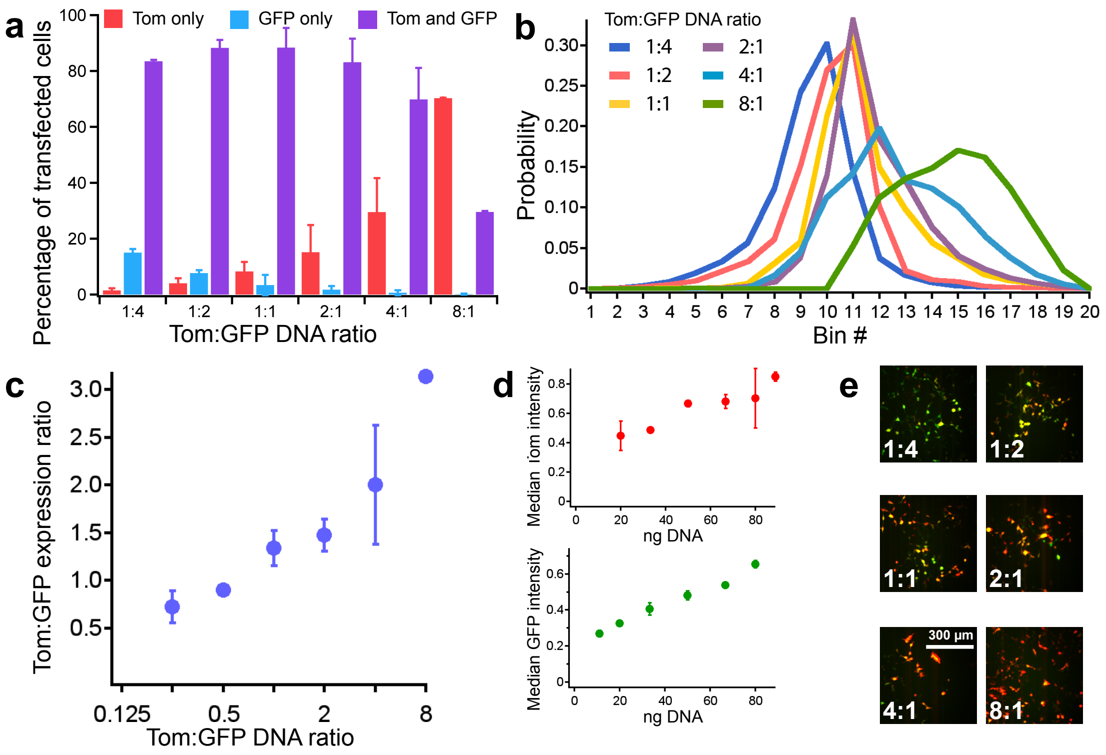

Supplementary Figure 12. Ratios of protein expression and transfection efficiency during co-transfection in a 96-well plate.
(a) Transfection efficiencies as a function of the Tom:GFP co-transfection ratio. Each sample represents the average from 2 wells (each imaged at 3 different positions) transfected with a specific Tom:GFP DNA ratio. Error bars show standard deviation. (b) Distribution of Tom:GFP expression ratios. Bin edges span from Tom:GFP expression ratios of 1/6 (left) to 6/1 (right), with increments of 1/0.5 or 0.5/1 (e.g. 1/6, 1/5.5, 1/5 … 5/1, 5.5/1, 6/1). Samples prepared as in (a). (c) Plot indicating the median Tom:GFP expression ratio from each sample shown in (b). Error bars show standard deviation. (d) Protein expression (measured by fluorescence intensity) as a function of the amount of DNA transfected. Samples were prepared as in (a). Error bars show standard deviation. (e) Sample images for each Tom:GFP co-transfection DNA ratio. Variance of the 96-well plate data from the on-chip data may be due to slightly different imaging conditions (on-chip exposure times: 50 ms Tom, 70 ms GFP; well plate exposure times: 50 ms Tom, 50 ms GFP).

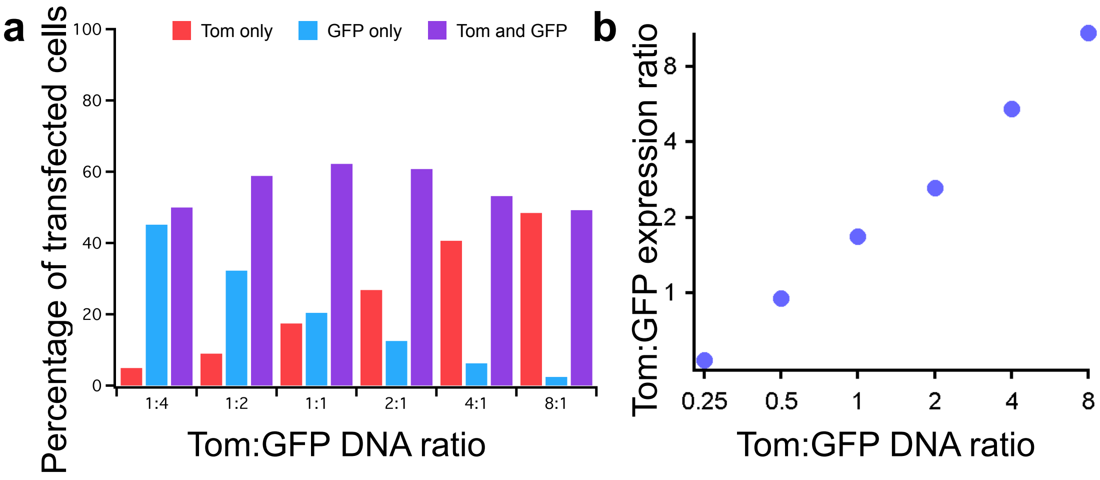

Supplementary Figure 13. Flow cytometry analysis for co-transfection in a 6-well plate.
(a) Transfection efficiencies as a function of the Tom:GFP co-transfection ratio. Each sample represents 50,000 cells transfected with a specific Tom:GFP DNA ratio. (b) Plot indicating the median Tom:GFP expression ratio from each co-transfection ratio shown in (a).

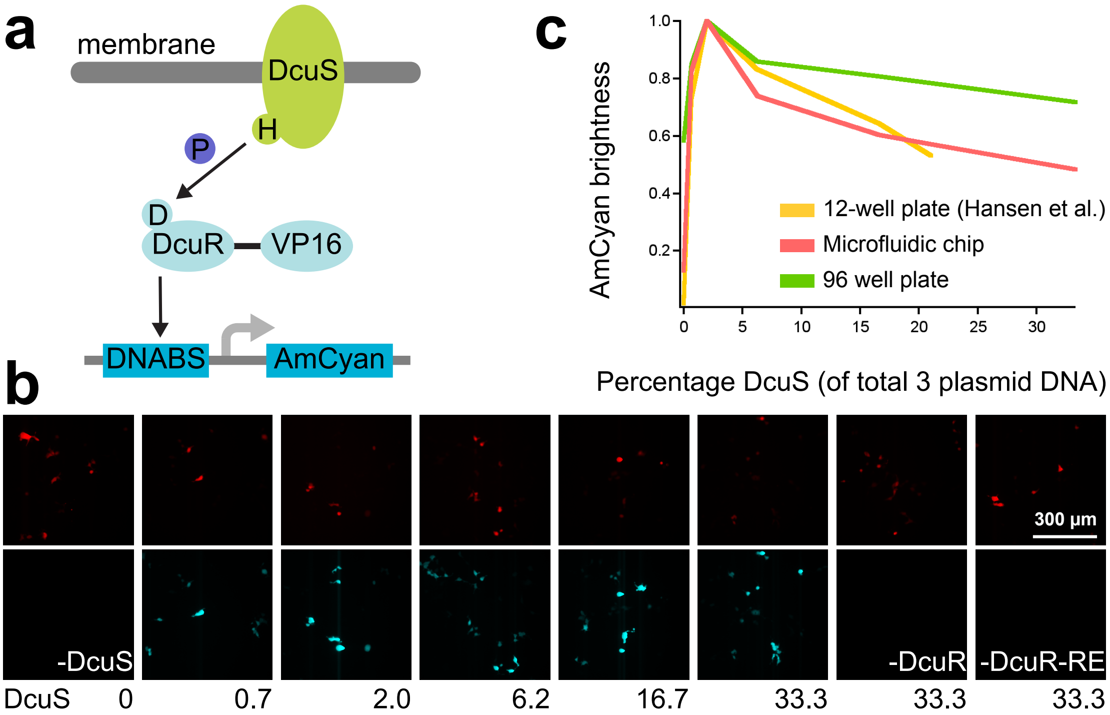

Supplementary Figure 14. Measuring the dynamics of synthetic gene circuits in a 96-well plate.
(a) Schematic of the two-component signaling pathway. The histidine kinase (DcuS) is activated by ligand binding and transmits the signal to DcuR, a DNA-binding protein. P, phosphate; VP16, VP16 transactivator domain, DNABS, DNA binding sites. (b) Representative fluorescence images with DcuS amounts indicated in ng. Each well of a 96-well plate was transfected with 13 ng tdTomato DNA and, aside from the negative controls, 38 ng DcuR and 56 DcuR-RE DNA. (c) Brightness of the reporter as a function of histidine kinase concentration for the original 12-well setup[32], for the microfluidic setup, and for the 96-well plate setup. For the microfluidic data, points are the average of 10 chambers. For the 96-well plate data, points are the average of 6 images originating from 2 wells. Brightness is normalized so that the maximum occurs at 1. Variance of the 96-well plate data from the on-chip data may be due to slightly different imaging conditions (on-chip exposure times: 100 ms Tom, 100 ms cyan; well plate exposure times: 50 ms Tom, 50 ms cyan).

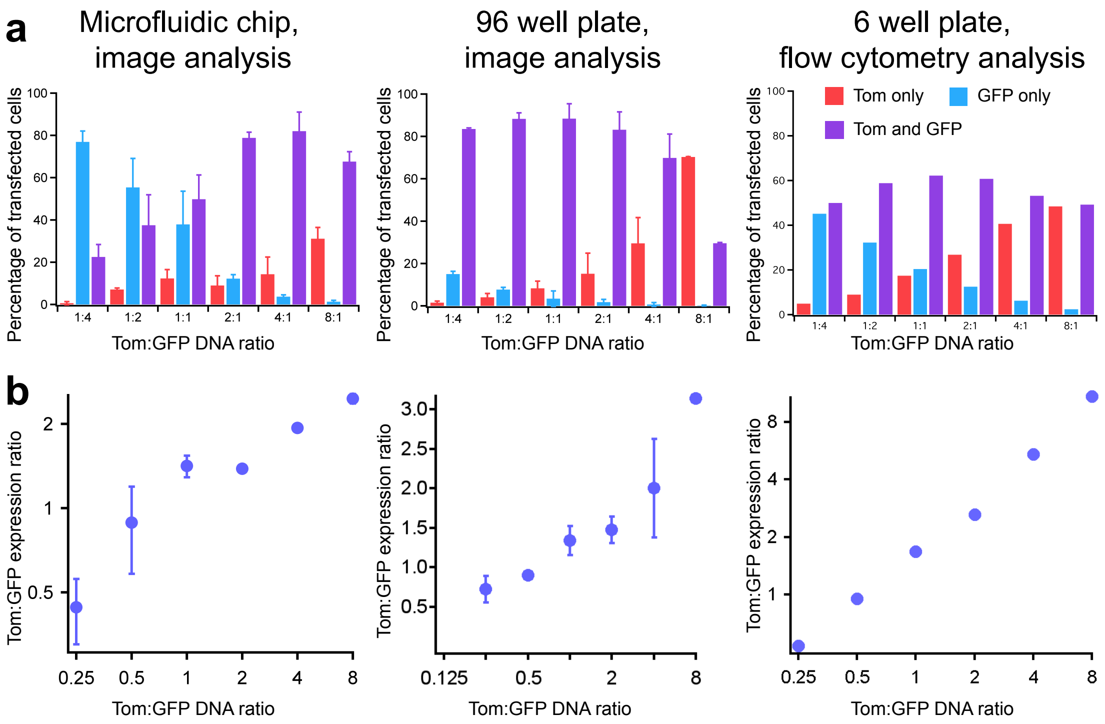

Supplementary Figure 15. Comparison of ratios of protein expression and transfection efficiency for various co-transfection and analysis methods.
(a) Transfection efficiencies as a function of the Tom:GFP co-transfection ratio, as previously shown in Fig. 6a, Supplementary Fig. 12a, and Supplementary Fig. 13a. (b) Plots indicating the median Tom:GFP expression ratio from each sample shown in (a).

Supplementary Table 1. Detailed composition of transfection mixtures used for optimization experiments.

Sample	DNA	Buffer	Effectene	Gelatin	Fibronectin	PLL
Fig. 2b	0.5 μg	6.5 μl EC buffer<br>1.2 μl 1.5 M sucrose<br>10 μl water	2 μl Enhancer<br>2 μl Effectene	6 μl, 1%	0	25 μl PLL + 50 μl water, spotted as indicated in figure
Fig. 2c	0.5 μg	15 μl EC buffer<br>4.5 μl 1.5 M sucrose	1.5 μl Enhancer<br>5 μl Effectene	11.85 μl, 0.5%	11.85 μl, 1 mg/ml	25 μl PLL + 50 μl of 0.15 or 0.225 M boric acid, pH 8.4 or 15 mM tris, pH 8.0, spotted at approximately 22 ng/mm^2
Fig. 2d	1.5 μg	15 μl EC buffer containing 0.2 M sucrose	1.5 μl Enhancer<br>5 μl Effectene	12.7 μl, 0.5%	12.7 μl, 1 mg/ml	25 μl PLL + 50 μl 0.225 M boric acid, pH 8.4, spotted at approximately 22 ng/mm^2
Fig. 3a	0.5 μg	15 μl EC buffer<br>4.5 μl 1.5 M sucrose	1.5 μl Enhancer<br>5 μl Effectene	23.7 μl, 0.5%	0	25 μl PLL + 50 μl 0.15 M boric acid, pH 8.4, spotted at approximately 22 ng/mm^2
Fig. 3b	0.5 μg	15 μl EC buffer<br>4.5 μl 1.5 M sucrose	1.5 μl Enhancer<br>5 μl Effectene	11.85 μl, 0.5%	11.85 μl, 1 mg/ml	25 μl PLL + 50 μl 0.15 M boric acid, pH 8.4, spotted at approximately 22 ng/mm^2
Fig. 3c	1.5 μg	15 μl EC buffer<br>4.5 μl 1.5 M sucrose	1.5 μl Enhancer<br>5 μl Effectene	11.85 μl, 0.5%	11.85 μl, 1 mg/ml	25 μl PLL + 50 μl 0.225 M boric acid, pH 8.4, spotted at approximately 22 ng/mm^2

Supplementary Table 2. Comparison of reagent requirements for various transfection methods. RT, reverse transfection.

	12 well plate	RT array (lipid-DNA)	Chip (lipid-DNA)	RT array (gelatin-DNA)	Chip (gelatin-DNA)
Effectene reagent (μl)	6	5	5	25 per array (1000s of reactions)	25 per chip (100s of reactions)
DNA (μg)	0.3	1.5	1.5	1.5	1.5

Supplementary Movie 1. Fluorescence time-lapse of tdTomato transfection.
The video represents the time period of 6-53 h post transfection, imaged approximately once per hour for tdTomato fluorescence. The sample contained 296 ng tdTomato, 59 ng DcuS, 444 ng DcuR 665 ng DcuR-RE.

Supplementary Movie 2. Fluorescence time-lapse of AmCyan transfection.
The same chamber was imaged as in Supplementary video 1, but for AmCyan fluorescence.

Supplementary Movie 3. Brightfield time-lapse of cell growth.
The same chamber was imaged as in Supplementary video 1, but in brightfield.
